# A case report of thoracic compartment syndrome in the setting of penetrating chest trauma and review of the literature

**DOI:** 10.1186/1749-7922-5-22

**Published:** 2010-07-30

**Authors:** Michael W Wandling, Gary C An

**Affiliations:** 1 Department of Surgery, Division of Trauma & Critical Care, Northwestern University Feinberg School of Medicine, 676 North Saint Clair St. Suite 6-650, Chicago, IL, 60611, USA; 2 Department of Surgery, Section of General Surgery, University of Chicago Pritzker School of Medicine, 5841 South Maryland, S-032 MC5031, Chicago, IL, 60637, USA

## Abstract

Trauma-related thoracic compartment syndrome (TCS) is a rare, life threatening condition that develops secondary to elevated intra-thoracic pressure and manifests itself clinically as significantly elevated airway pressures, inability to provide adequate ventilation and hemodynamic instability temporally related to closure of a thoracic surgical incision. TCS is exceedingly rare in the trauma population. We present a case of TCS following surgical repair of a stab wound injury that necessitated decompressive thoracotomy and peri-operative open-chest management.

## Background

While abdominal compartment syndrome is a well-recognized clinical entity in the trauma population, the thoracic cavity is a significantly less frequent site of compartment syndrome. Thoracic compartment syndrome (TCS) has been primarily reported in relation to cardiac/mediastinal procedures [1-5]. Although TCS has been reported outside of the cardiac surgery population, it is exceedingly rare in the trauma population and no case has been reported without cardiac involvement. Here, we present a case of TCS where initiation and pathogenesis were entirely non-cardiac in origin following surgical repair of a stab wound injury that necessitated decompressive thoracotomy and peri-operative open-chest management.

## Case Presentation

**Figure 1:**
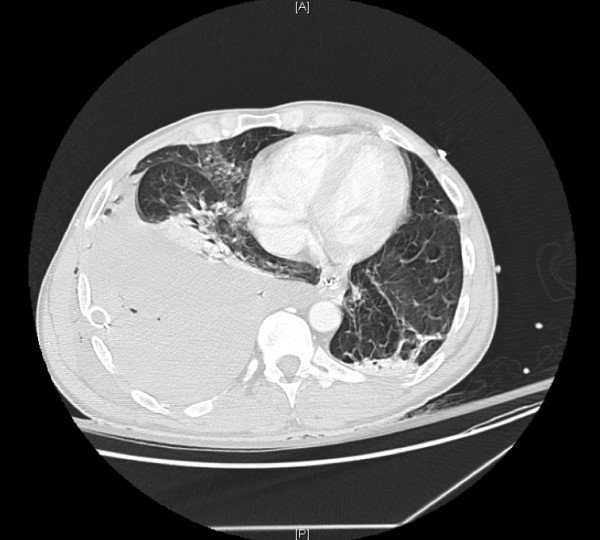
**CTA of chest revealing large residual clot in the right hemi-thorax**. This study was performed in an attempt to localize the bleeding source in our patient. The study was negative in terms of identifying an anatomic source of bleeding (most relevant with respect to examination of the great vessels in the thoracic outlet, albeit falsely negative). However, this study served as a proxy for the post-thoracostomy chest x-ray and identified the insufficient drainage of the right chest with the thorocostomy tube in place.

A 46-year-old male was brought to the emergency department at Northwestern Memorial Hospital with multiple stab wounds to the neck and chest. He was hypotensive upon arrival and a right needle thoracostomy returned blood and air, resulting in improvement in blood pressure. Secondary survey demonstrated a stab wound to Zone I of the right neck, approximately 2 cm above the right clavicular head, and a second stab wound to the right thoraco-abdominal area 3 cm above the costal margin and 2.5 cm lateral to the mid-clavicular line. A portable chest x-ray performed at Patient Arrival Time (PAT) + 10 min revealed a right hemothorax. A right thoracostomy tube was placed, which returned 800 mL of blood. By this time the patient had responded to resuscitation of 2 L of Lactated Ringers (PAT + 20 min). The patient did not at this time meet criteria for an emergent thoracotomy (< 1500 mL thoracostomy output and hemodynamic stability), therefore planning the workup for potential surgical sources of bleeding incorporated 3 areas of concern: 1) intra-thoracic injury resulting from the lower right thoraco-abdominal wound, 2) intra-abdominal injury from the lower right thoraco-abdominal wound that was decompressing through a diaphragm injury into the right thoracic cavity and 3) injury to the proximal great vessels from the Zone I neck wound decompressing into the right thoracic cavity. We believed that distinguishing between these three possibilities was important in so far that the optimal surgical approach to each area was different: 1) posterior thoracotomy for thoracic injury, 2) laparotomy for abdominal and 3) median sternotomy/clavicular extension for proximal great vessel exposure. A focused abdominal sonogram for trauma (FAST) done at PAT + 20 min was negative. Given the range of possible injuries and the patient's current stability, a Computer Tomography Angiogram (CTA) of the neck and chest and a CT scan of the abdomen were performed at PAT + 40 min. Although no contrast extravasation suggestive of active bleeding was appreciated on CT, a residual clot occupying the > 50% of the right chest was appreciated (see Figure 1). There was no evidence of intra-abdominal injury on the CT scan of the abdomen. A second thoracostomy tube was placed and approximately 2.2 L of blood were evacuated with suction. Given that this output now met criteria for surgical exploration, the decision was made to take the patient to the operating room for an exploratory thoracotomy (PAT + 60 min). Resuscitation up to this point consisted of 4 L of crystalloid and 6 units of PRBCs.

As a bleeding source had not yet been identified, all three potential areas of injury remained viable concerns. Given this uncertainty, the decision was made to utilize the surgical approach that would provide the greatest flexibility for our set of potentialities. Our opinion was that a right antero-lateral thoracotomy provided the best trade-off between flexibility and exposure, and this was performed at the sixth interspace at PAT + 80 min. Exploration revealed approximately 2 L of blood and clot, a hematoma in the right superior mediastinum overlying the origin of the great vessels, and a wound in the pleura in this area that was not initially bleeding, but developed pulsatile arterial and dark venous bleeding during exploration. Given the diagnosis of injury to the right great vessels, the antero-lateral thoracotomy was converted to a trap-door incision in order to facilitate exposure of this area. A through and through injury to the proximal right subclavian vein was identified, and with further exposure, a second injury was identified involving a transection of the right internal mammary artery approximately 1 cm from its origin from the right subclavian artery. Due to hypothermia and coagulopathy, subclavian vein reconstruction was deferred and the vein was ligated. The internal mammary artery was ligated as well. Due to coagulopathy, the decision was made to pack the right chest for hemostasis and place topical hemostatic agents over the areas of dissection and at the edges of the thoracotomy. Definitive chest closure was deferred and only the skin was closed over the trap-door incision, while leaving two thoracostomy tubes in place. Following closure, the patient was noted to have high airway pressures and a tense abdomen, consistent with abdominal compartment syndrome (ACS). Given these clinical features in the presence of ACS risk factors (massive ongoing fluid resuscitation), formal measurement of intra-abdominal pressure was deferred and a midline decompressive laparotomy was performed, resulting in the patient's airway pressures rapidly declining from 50 cmH_2_O to 40 cmH_2_O with improvement of oxygenation and hemodynamic status. A Bogota bag was sewn onto the skin surrounding the abdominal incision and Jackson-Pratt drains were placed at the superior and inferior aspects. The total time of the procedure was 156 minutes with an estimated blood loss of 17 L. In the operating room, the patient received 49 units of packed red blood cells, 12 units of fresh frozen plasma, 3 units of cryoprecipitate, 3 units of platelets and Factor VII. Prior to leaving the operating room, the patient was hypothermic with a core temperature of 31°C, but relatively hemodynamically stable and not supported by pressors.

**Figure 2:**
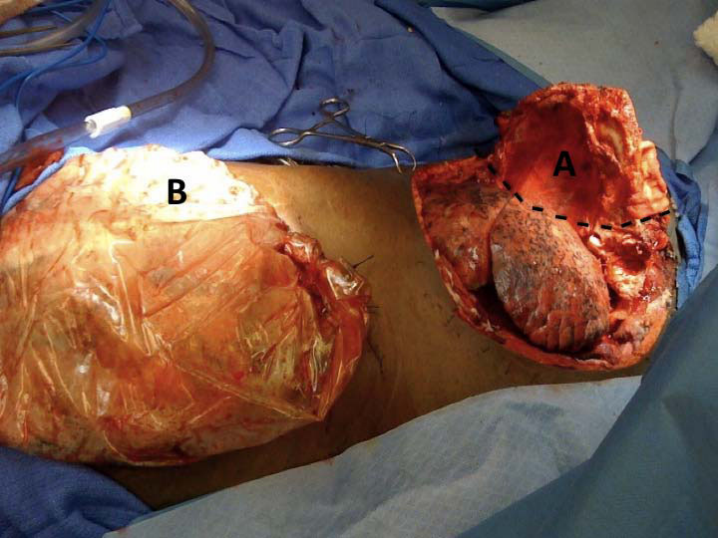
**Status of patient's wounds upon return to the operating room after 24 hours of open-chest management**. The development of thoracic compartment syndrome necessitated therapeutic re-opening of the chest and open-chest management. A) Open trap-door thoracotomy. Comprised of connecting anterolateral thoracotomy in the 6^th ^intercostal space, partial sternotomy, and supraclavicular incisions. The reflection edge for the trap-door is shown by the black hatched lines: the ribs along this edge were fractured by the reflection of the trap-door. B) Open midline laparotomy with Bogota bag sewn onto the skin.

Upon arrival to the surgical intensive care unit, approximately at post-operative time (POT) + 30 minutes, the patient had another elevation in airway pressure, with an inability to deliver adequate tidal volumes via the ventilator and profound hypotension. Both chest tubes appeared to be functioning. The patient could be manually bagged, but with very high resistance. At that time it was believed that increased pressure in the right chest was impairing the ability to expand the right lung and also compromising cardiac function; all findings consistent with a thoracic compartment syndrome. The decision was made at POT + 50 minutes to re-open the trap-door incision in the intensive care unit to release the pressure and evaluate for ongoing surgical bleeding. Upon reopening the right chest there was immediate improvement in ventilation and blood pressure with approximately 1 L of clot present. Exploration of the chest cavity did not demonstrate surgical bleeding, though all dissection planes were oozing. The chest was repacked, and due to the prior episode of life-threatening ventilatory and hemodynamic compromise, the decision was made to manage the patient with an open chest cavity to allow for respiratory and hemodynamic stabilization while correcting the hypothermia and coagulopathy. An adhesive plastic drape was folded over (to remove the adhesive surface) and placed over the right lung and a second adhesive plastic drape was placed over the entire trap-door incision to close the pleural space. The plastic drape was then vented medially to prevent the development of a tension pneumothorax. The patient stabilized and responded to rewarming and correction of his coagulopathy. At ~POT + 30 hours the patient was returned to the operating room for removal of chest packing and chest closure. Figure 2 demonstrates the status of the patient's wounds at time if initial return to the operating room. The chest was too tight to undergo a definitive sternal and pericostal closure, so soft-tissue closure was once again obtained by running the skin closed along the perimeter of the trap-door. Abdominal closure was deferred to the time of definitive chest closure, both of which were performed five days later.

The patient had an extensive treatment course in the surgical intensive care unit, manifesting severe acute respiratory distress syndrome, requiring inhaled nitric oxide and prone-positioning ventilation. The patient also developed acute renal failure and severe deconditioning. The patient was eventually discharged to a long-term ventilatory care facility on post-trauma day 68, and returned to his home approximately 2 months thereafter.

## Discussion

Thoracic compartment syndrome (TCS) has been reported predominantly in the pediatric and adult cardiac surgery populations, where this phenomenon has been described as a syndrome of "mediastinal tightness" following prolonged cardiac surgery [2-5]. In this setting, post-operative myocardial edema, acute ventricular dilatation and pulmonary and chest wall edema raises mediastinal pressure, leading to compression of the heart, decreased cardiac output, decreased diastolic filling and hemodynamic collapse in a process similar to cardiac tamponade. This often develops during or immediately following sternal re-approximation, however, it may not develop for hours or even days after chest closure [2-6].

TCS secondary to trauma is exceedingly rare. A review of the literature revealed only one prior report of TCS in the setting of trauma. In that report, Kaplan et al [1] presented a case of a patient with gunshot wounds through the heart and descending thoracic aorta who developed TCS upon clamshell thoracotomy closure. In that case, closure of the chest precipitated an immediate elevation in airway pressure and rapid hemodynamic collapse. Given the extent of his injuries and the incision used, it would be reasonable to consider both of his pleural spaces and his mediastinum as one contiguous space, and that the development of TCS likely affected all thoracic structures equally.

Intensive resuscitative and surgical measures are not uncommon in trauma surgery, yet the development of TCS is extremely rare. We believe that some of the challenges associated with our patient may have contributed to the development of TCS. We have identified certain points that we believe merit increased discussion.

1) Prolonged pre-operative period: Our patient had an hour of pre-operative management during which he had a surgically amenable injury. In many ways, our patient typifies the dilemma of the "meta-stable" trauma patient: that patient who responds to initial resuscitative measures yet for whom there remains significant concern that surgical intervention will be necessary. As described, this patient did not meet the criteria for immediate thoracotomy based on chest tube output (< 1500 mL of initial output), however this evaluation was confounded by the fact that the thoracostomy tube was clotted. Reliance upon the chest tube output is predicated upon fully expanding the lung; this was not the case in our patient. A repeat chest x-ray would have prompted another chest tube (the course of action that in our case followed the chest CT); therefore, had a chest x-ray been done prior the chest CT (a time interval of 20 minutes) then the criteria for an immediate thoracic exploration would have been met and the patient would have been taken to the operating room approximately 30 minutes earlier. It is possible to infer that that delay may have contributed to the degree of ischemia-reperfusion injury associated with hemorrhage, though as noted, our patient had an appearance of stability and cessation of bleeding during this period of time resulting from temporary tamponade of the vascular injury within the mediastinal hematoma. In either case, uncertainty regarding the surgical approach (unfortunately not addressed by the CT scan, the purpose for which it was performed) was present.

2) Determination of surgical approach: The classical approach to traumatic intra-thoracic bleeding is via a postero-lateral thoracotomy. However, the exception to this is when there is concern for a concurrent intra-abdominal injury or a right-sided thoracic outlet injury; exposure to both of these areas are significantly limited in the lateral decubitus position required for a postero-lateral approach. The recommended exposure for proximal subclavian injuries is via a median sternotomy or clavicular resection [7,8], best accomplished with the patient supine. Therefore, the decision hinged upon which represented the best compromise: attempting to address a thoracic injury via an anterior approach, or attempting to deal with potential mediastinal or abdominal injuries in a patient in lateral decubitus position. We selected the supine approach with the rationale that this provided the best compromise given the range of possible injuries. Therefore, the initial incision would reasonably be an antero-lateral thoracotomy to best delineate the actual source of bleeding, which was accomplished.

3) Pathogenesis of elevated intra-thoracic pressure: Our patient was at risk for elevated thoracic cavity pressures due to space-occupying hemostatic packing of the pleural space and decreased compliance of the chest wall secondary to increased edema from systemic resuscitation and direct tissue trauma. However, in most circumstances neither situation alone would have precipitated a TCS, as the amount of packing in the chest amounted to only approximately 1 L worth of clot, and the amount of resuscitation was, while considerable, not unheard of. We believe that a significant contributing factor was the decreased chest wall compliance secondary to the substantial tissue injury accompanying the trap-door thoracotomy. The trap-door needed to be reflected laterally to gain exposure, breaking the ribs involved (see Figure 2). The direct tissue trauma and degree of systemic resuscitation resulted in greater amounts of chest wall edema than would normally be experienced. Decompressive thoracotomy, through reopening of the trap-door incision, allowed free expansion of the right lung with consequent improvement in ventilation, respiratory acidosis and cardiac function.

4) Open-chest management: Given the improvement in respiratory function following reopening of the chest, we decided that it would have been unwise to attempt re-closure of the chest wound. In the cardiac surgery literature, prevention and treatment of TCS rely on reduction of intra-thoracic pressure and delayed sternal closure [2-6]. Management techniques range from loose closure with synthetic materials or skin flaps to leaving the chest open and packed [2]. In the case presented by Kaplan et al [1], open chest management was reported, where the chest was packed and covered with a sterile, occlusive, water impermeable drape. We followed a similar approach, where after hemostatic packing, the lung was covered with a non-adhesive plastic sheet to allow for lung movement with ventilation and the surgical wound was covered with an adhesive plastic drape and vented medially to prevent the development of a tension pneumothorax. In terms of the timing for return to the operating room, we followed the same general guidelines as with a damage control laparotomy: as soon as the patient had been re-warmed and the coagulopathy corrected the patient was taken back to the operating room for removal of packing and an attempt at definitive closure.

## Conclusion

Thoracic compartment syndrome is a rare, but life-threatening phenomenon in trauma patients following massive resuscitation. Concurrent chest wall trauma, either primary or due to surgical exposure, and the need for intra-thoracic hemostatic packing represent additional risk factors. The clinical characteristics of TCS are significantly raised airway pressures, inability to provide ventilation and hemodynamic instability. Since abdominal compartment syndrome is a much more common cause of elevated airway pressures in trauma patients, it should be ruled out before making the diagnosis of TCS. Development of symptoms of TCS, particularly during or shortly after chest closure, should prompt immediate chest decompression and open chest management until hypothermia, acidosis and coagulopathy are corrected and hemodynamic stability is attained.

## Competing interests

The authors declare that they have no competing interests.

## Authors' contributions

GA participated in the care of this patient, manuscript preparation and literature search. MW participated in manuscript preparation and literature search. GA and MW co-authored the writing of the manuscript. Both authors read and approved the final manuscript.

## Consent

Written informed consent was obtained from the patient for publication of this case report and any accompanying images. A copy of the written consent is available for review by the Editor-in-Chief of this journal.

## References

[B1] Kaplan LJ Trooskin SZ Santora TA Thoracic compartment syndrome J Trauma 1996 40 2 291 3 10.1097/00005373-199602000-00021 8637082

[B2] Rizzo AG Sample GA Thoracic compartment syndrome secondary to a thoracic procedure: a case report Chest 2003 124 3 1164 8 10.1378/chest.124.3.1164 12970052

[B3] Alexi-Meskishvili V Prolonged open sternotomy after pediatric open heart operation: experience with 113 patients Ann Thorac Surg 1995 59 2 379 83 10.1016/0003-4975(94)00840-4 7847952

[B4] Christenson JT Open chest and delayed sternal closure after cardiac surgery Eur J Cardiothorac Surg 1996 10 5 305 11 10.1016/S1010-7940(96)80087-X 8737685

[B5] Riahi M Cardiac compression due to closure of the median sternotomy in open heart surgery Chest 1975 67 1 113 4 10.1378/chest.67.1.113 1235315

[B6] Amato J Review of the rationale for delayed sternal closure Crit Care Med 2000 28 4 1249 51 10.1097/00003246-200004000-00072 10809329

[B7] Buscaglia LC Walsh JC Wilson JD Matolo NM Surgical management of subclavian artery injury Am J Surg 1987 154 1 88 92 10.1016/0002-9610(87)90295-9 3605517

[B8] Demetriades D Chahwan S Gomez H Peng R Velmahos G Murray J Asensio J Bongard F Penetrating injuries to the subclavian and axillary vessels J Am Coll Surg 1999 188 3 290 295 10.1016/S1072-7515(98)00289-0 10065818

